# Supplementary material for: Ensemble inference of unobserved infections in networks using partial observations
Source: PLoS Comput Biol. 2023 Aug 7;19(8):e1011355. doi: 10.1371/journal.pcbi.1011355 (PMC10434926; doi:10.1371/journal.pcbi.1011355)
Supplement: S1 Text — Extended methods and analyses. (PDF) [file pcbi.1011355.s001.pdf]

Supplementary material

Epidemic model

For an undirected network, each node is in one of three states: susceptible (S), infected (I) and recovered (R). A susceptible individual i can be infected by an infected neighbor with a transmission rate β_i on each day, independent of the states of other neighbors. An infected individual recovers with a probability $1/D$, where D is the average infectious period. In all experiments, we fixed $D = 7$ days. To represent variations in transmission rates, β_i was drawn from a bimodal distribution, created by superimposing two Gaussian distributions (Fig A). A power-law distribution for transmission rate ($P(\beta) \propto \beta^{-\gamma}, \gamma = 2.5$) was also tested in additional experiments.

A set of seeds were selected to initiate outbreaks in a fully susceptible population. Seeds, transmission rate distribution and simulation time were selected to produce outbreaks that infect 25-35% population by the end of simulation (i.e., the infected (I) and recovered (R) individuals account for 25-35% population on the last day). We simulated outbreaks for 7 days in networks with less than 5,000 nodes, and 14 days in larger networks that need more time to infect the target percentage of population. We chose this epidemic regime for a meaningful comparison of inference algorithms – if the percentage of infected population is very high (low), inference that estimates all (none) individuals are infected would produce an artificial high accuracy. In addition, this epidemic regime represents the early to middle stage of outbreaks, when accurate inference is useful for real-time disease control.

We modeled the observational process using daily testing probabilities for people in different states. On each day, each node is randomly tested with a probability depending on the state. We assume infected persons are more likely to be tested than susceptible and recovered persons. The states of tested individuals (S, I or R) are obtained on various testing dates. By varying daily testing probabilities, we can adjust the percentage of observed individuals. In the experiments, we simulated observations of around 15%, 30% and 50% of the total population. Detailed configurations of epidemic models, including distribution of transmission rate and testing probability, are provided in Table A.

Inference framework

The evolution of $P(X_i^t)$ ($X \in (S, I, R)$) can be described by a set of master equations:

$$\frac{dP(S_i^t)}{dt} = -P(S_i^t)\left[1 - \prod_{j\in\partial i}\left(1 - \beta_i P\left(I_j^t\right)\right)\right], \quad \text{(S1)}$$

$$\frac{dP(I_i^t)}{dt} = P(S_i^t)\left[1 - \prod_{j\in\partial i}\left(1 - \beta_i P\left(I_j^t\right)\right)\right] - \frac{P(I_i^t)}{D}, \quad \text{(S2)}$$

$$\frac{dP(R_i^t)}{dt} = \frac{P(I_i^t)}{D} \ , \tag{$S3$}$$

Here, β_i is the transmission rate of node i, ∂i is the set of neighbors of node i, and D is the average infectious duration. Equations (1-3) are accurate when the network has a tree structure. For real-world networks without too many short loops, previous studies found Equations (1-3) provide a good approximation [1]. An ensemble of $P(X_i^t)$ was initiated at time $t = 0$ by randomly drawing $P(I_i^0)$ from a uniform distribution and setting $P(S_i^0) = 1 - P(I_i^0)$ and $P(R_i^0) = 0$. The transmission rate β_i for each node was drawn from a uniform distribution and was fixed during model simulation.

To estimate $P(X_{i_o}^t | X_{i_o}^{t_{i_o}} = O)$ $(t_{i_o} > t)$, we use a Bayesian approach:

$$P\left(X_{i_o}^t \middle| X_{i_o}^{t_{i_o}} = O\right) \propto P\left(X_{i_o}^t\right) \times P\left(X_{i_o}^{t_{i_o}} = O \middle| X_{i_o}^t\right), \tag{S4}$$

where $P\left(X_{i_o}^t\right)$ is the prior probability and $P\left(X_{i_o}^{t_{i_o}} = O \middle| X_{i_o}^t\right)$ is the likelihood of observing state O of individual i_o at time t_{i_o} given the state $X_{i_o}^t$ at time t. In the sequential inference algorithm, the prior $P\left(X_{i_o}^t\right)$ can be obtained by integrating the posterior estimates of the probabilities of i_o's states at time $t - 1$ to time t. The likelihood $P\left(X_{i_o}^{t_{i_o}} = O \middle| X_{i_o}^t\right)$ can be computed using the master equations in which the state of i_o at time t is fixed as S, I, or R. We use the normalization condition to calculate the posterior $P\left(X_{i_o}^t \middle| X_{i_o}^{t_{i_o}} = O\right)$ for $X_{i_o}^t = S, I$ and R. More details are provided in the following section.

Theoretically, it is ideal to estimate the probability of $X_{i_o}^t$ given all future observations, i.e., $P\left(X_{i_o}^t \middle| \left\{X_j^{t_j}\right\}, t_j > t\right)$, as the states of nodes in the network may be interdependent. However, this approach requires to run master equations for each observed individual i_o separately to compute the likelihood $P\left(\left\{X_j^{t_j}\right\}, t_j > t \middle| X_{i_o}^t\right)$, which can be computationally prohibitive for large networks with a large number of observations. For randomly observed sparse observations, we can assume the interdependency of their states is negligible and estimate the posterior of i_o, $P(X_{i_o}^t | X_{i_o}^{t_{i_o}} = O)$, without considering observations of other nodes. This simplification can dramatically speed up the computation of the likelihood $P\left(X_{i_o}^{t_{i_o}} = O \middle| X_{i_o}^t\right)$: if observed individuals are independent, we can calculate the likelihood for all observed nodes simultaneously. In synthetic tests, we found that the simplified algorithm yields comparable performance as the algorithm considering interdependency of observed nodes (Figs B, D, E, F, G).

To check the validity of the relative independency of observations, we performed an analysis to examine the distance between observed individuals in the contact network. We computed the distribution of the pairwise shortest distance between observed nodes in three real-world networks – Slashdot, Twitter, and Digg (Fig I). On average, the distance between most pairs of

observations is larger than 2 steps. For a small transmission rate, we can assume the observed nodes are relatively independent.

Pseudo-code and implementation

The pseudo-code for the ensemble inference algorithm is provided below. Line 12, model integration, can be performed by running the master equations forward in time.

Input: Master equations M, an ensemble (K members) of initial probabilities $P(X_i^0)$ for all nodes i for $X = S, I$ and R, observations $\mathcal{D} = \{(i_o, t_o, O)\}$ during time 1 and T.

---

1 For $t = 1$ to T

2 **For** each node i_o observed after time t

3 **For** each ensemble member k = 1 to K

4 Update $P(S_{i_o}^t)$, $P(I_{i_o}^t)$ and $P(R_{i_o}^t)$ using observation at time t_o, (i_o, t_o, O)

5 **End For**

6 **For** each neighbor j of i_o

7 **For** each ensemble member k = 1 to K

8 Update $P(S_j^t)$, $P(I_j^t)$ and $P(R_j^t)$ using covariability adjustment

9 **End For**

10 **End For**

11 **End For**

12 Compute $P(S_i^{t+1})$, $P(I_i^{t+1})$ and $P(R_i^{t+1})$ for all individuals by integrating M to time $t + 1$

13 End For

Output: $P(S_i^T)$, $P(I_i^T)$ and $P(R_i^T)$ for all individuals at time T

Backward temporal propagation

Here, we provide more details to perform line 4 in the algorithm (i.e., the backward temporal propagation of information). To estimate $P(X_{i_o}^t | X_{i_o}^{t_{i_o}} = O)$ $(t_o > t)$ for each ensemble member, we need to use the prior $P\left(X_{i_o}^t\right)$ and the likelihood $P\left(X_{i_o}^{t_{i_o}} = O \middle| X_{i_o}^t\right)$. We can use the current

estimate of $P\left(X_{i_o}^t\right)$ before the update at time t as the prior. The major computational task is to estimate the likelihood $P\left(X_{i_o}^{t_{i_o}} = O \middle| X_{i_o}^t\right)$. For instance, to compute $P\left(X_{i_o}^{t_{i_o}} = O \middle| X_{i_o}^t = S\right)$ (that is, the probability of observing the state of i_o at time t_{i_o} is O given that i_o is susceptible at time t), we can set $P\left(X_{i_o}^t = S\right) = 1$ in the master equations at time t (keep the probabilities of other nodes as the current estimates) and run the master equations from time t to t_{i_o}. The likelihood $P\left(X_{i_o}^{t_{i_o}} = O \middle| X_{i_o}^t = S\right)$ can be directed obtained as the probability of node i_o in state O at time t_{i_o} in the master equations. Similarly, we can obtain the likelihood estimates $P\left(X_{i_o}^{t_{i_o}} = O \middle| X_{i_o}^t = I\right)$ and $P\left(X_{i_o}^{t_{i_o}} = O \middle| X_{i_o}^t = R\right)$. Once we have the likelihood, we can compute $P\left(X_{i_o}^t\right) \times P\left(X_{i_o}^{t_{i_o}} = O \middle| X_{i_o}^t = S\right)$, $P\left(X_{i_o}^t\right) \times P\left(X_{i_o}^{t_{i_o}} = O \middle| X_{i_o}^t = I\right)$ and $P\left(X_{i_o}^t\right) \times P\left(X_{i_o}^{t_{i_o}} = O \middle| X_{i_o}^t = R\right)$. Finally, using the normalization condition $P\left(X_{i_o}^t = S \middle| X_{i_o}^{t_{i_o}} = O\right) + P\left(X_{i_o}^t = I \middle| X_{i_o}^{t_{i_o}} = O\right) + P\left(X_{i_o}^t = R \middle| X_{i_o}^{t_{i_o}} = O\right) = 1$, we can calculate the posterior $P\left(X_{i_o}^t \middle| X_{i_o}^{t_{i_o}} = O\right)$.

To update $P\left(X_{i_o}^t\right)$ at time t, it is more accurate to estimate the probability of $X_{i_o}^t$ given all future observations i.e., $P\left(X_{i_o}^t \middle| \left\{X_j^{t_j}\right\}, t_j > t\right)$, as the states of nodes in the network may be interdependent. This approach has been implemented in a previous study but is computationally expensive as the likelihood involves the states of multiple nodes and the computation needs to be performed for each observed individual separately. The high computational cost has limited the application of the inference algorithm to networks of size $O(10^3)$. In this study, we introduced a simplification assuming the interdependency of sparse observations is negligible. As a result, we can estimate the posterior of i_o, $P(X_{i_o}^t | X_{i_o}^{t_{i_o}} = O)$, without considering observations of other nodes. This simplification can dramatically speed up the computation of the likelihood.

In order to compute the likelihood $P\left(X_{i_o}^{t_{i_o}} = O \middle| X_{i_o}^t\right)$ for all observed individuals, the straightforward implementation is to run the master equations for each observed individual i_o separately. In our implementation, we used an approximation that can compute $P\left(X_{i_o}^{t_{i_o}} = O \middle| X_{i_o}^t\right)$ for all observed individuals simultaneously by running the master equations only once for $X_{i_o}^t = S$, I and R. Assuming the states of sparsely observed individuals are relatively independent, we can set $P\left(X_{i_o}^t = S\right) = 1$ (or $P\left(X_{i_o}^t = I\right) = 1$, $P\left(X_{i_o}^t = R\right) = 1$) for all observed individuals at time t and run the master equations from time t to the time of latest observation. If the states of observed individuals are not strongly dependent, we can approximate $P\left(X_{i_o}^{t_{i_o}} = O \middle| X_{i_o}^t\right)$ using the results of the master equations at time t_{i_o} for each i_o.

Here we highlight the difference between the simplified algorithm (ENS-I) and the full inference algorithm (ENS-I O) that considers the interdependency of observed individuals. In the full inference algorithm ENS-I O, to compute the likelihood $P\left(X_{i_o}^{t_{i_o}} = O \middle| X_{i_o}^t\right)$, we need to run the master equations for each observation $X_{i_o}^t$. Specifically, for each observation $X_{i_o}^t$, we set $P\left(X_{i_o}^t = S\right) = 1$ at time t while keeping the states of other nodes unchanged, run the master

equations till time t_{i_o}, and obtain $P\left(X_{i_o}^{t_{i_o}}=O \middle| X_{i_o}^t=S\right)$. We need to additionally run master equations for $P\left(X_{i_o}^t=I\right)=1$ and $P\left(X_{i_o}^t=R\right)=1$ to get the posterior distribution. If there are N_o observations, we need to run the master equations for $3N_o$ times. In contrast, in the simplified algorithm ENS-I, we want to compute $P\left(X_{i_o}^{t_{i_o}}=O \middle| X_{i_o}^t\right)$ for all observed individuals simultanesously. To do this, at time t, we set $P\left(X_{i_o}^t=S\right)=1$ for all observed nodes i_o, run the master equations, and obtain $P\left(X_{i_o}^{t_{i_o}}=O \middle| X_{i_o}^t=S\right)$ for all i_o. Following the same method, we can compute $P\left(X_{i_o}^{t_{i_o}}=O \middle| X_{i_o}^t=I\right)$ and $P\left(X_{i_o}^{t_{i_o}}=O \middle| X_{i_o}^t=R\right)$ for all i_o. As a result, we only need to run the master equations for 3 times for all N_o observations. The assumption for the simplified algorithm ENS-I is that the states of observations are relatively independent, so changing the states of other observed nodes won't impact much of the states of the focal nodes. Given the sparse observation and the distance between observed nodes, this assumption is reasonable.

We performed a comparison between the simplified algorithm and the full inference algorithm. For a range of network structures and the percentage of observed individuals, the simplified algorithm yielded similar performance as the full inference algorithm (Figs B, D, E, F, G). Therefore, we presented results obtained from the simplified algorithm in the main text.

Cross-ensemble covariability adjustment

The detail of line 8 (i.e., cross-ensemble covariability adjustment) is provided here. We update the probabilities of observed individuals' neighbors using cross-ensemble covariability. Specifically, define $\Delta P(X_{i_o}^t)$ as the adjustment on the prior probability $P(X_{i_o}^t)$ in the last procedure: $\Delta P\left(X_{i_o}^t\right)=P\left(X_{i_o}^t \middle| X_{i_o}^{t_{i_o}}=O\right)-P\left(X_{i_o}^t\right)$. For each neighbor j of individual i_o, we adjust $P(X_j^t)$ by adding

$$\Delta P\left(X_j^t\right)=\frac{cov\left(P\left(X_j^t\right),P\left(X_{i_o}^t\right)\right)}{var\left(P\left(X_{i_o}^t\right)\right)}\times\Delta P\left(X_{i_o}^t\right) \qquad (S5)$$

to the prior $P(X_j^t)$. Here, $cov\left(P\left(X_j^t\right),P\left(X_{i_o}^t\right)\right)$ is the cross-ensemble covariance between $P\left(X_j^t\right)$ and $P\left(X_{i_o}^t\right)$, and $var\left(P\left(X_j^t\right)\right)$ is the ensemble variance of $P\left(X_j^t\right)$. Both quantities can be directed computed using ensemble members. The adjusted $P(X_j^t)$ is set as $P\left(X_j^t\right)+\Delta P(X_j^t)$. This update procedure has been used in the ensemble adjustment Kalman filter (EAKF), an efficient Bayesian inference algorithm, to preserve high-order moments of the prior distribution [2]. To guarantee the normalization condition ($P\left(S_j^t\right)+P\left(I_j^t\right)+P\left(R_j^t\right)=1$), we normalize the adjusted $P(X_j^t)$ for $X=S, I$ and R.

The modified DMP algorithm

The dynamic message-passing (DMP) algorithm was initially developed to address the question of inferring the origin of an epidemic following the SIR model in networks. Specifically, DMP uses a set of dynamic message-passing equations to describe the evolution of probabilities of individuals in each state (S, I, or R). Define the cavity message $\theta^{k\to i}(t)$ as the probability that the infection signal has not been passed from node k to node i up to time t when node i is fixed to the state S, and , $\phi^{k\to i}(t)$ is the probability that the infection signal has not been passed from node k to node i up to time t when node i is fixed to the state S and node k is in the state I at time t. The update rules for cavity messages are

$$P_S^{i\to j}(t+1) = P_S^i(0)\prod\nolimits_{k\in\partial i\backslash j}\theta^{k\to i}(t+1), \qquad (S6)$$

$$\theta^{k\to i}(t+1) - \theta^{k\to i}(t) = -\lambda_{ki}\phi^{k\to i}(t), \qquad (S7)$$

$$\phi^{k\to i}(t) = (1-\lambda_{ki})(1-\mu_k)\phi^{k\to i}(t-1) - \left[P_S^{k\to i}(t) - P_S^{k\to i}(t-1)\right]. \qquad (S8)$$

Here λ_{ki} is the transmission probability from node k to node i, μ_k is the recovery probability of node k, $P_S^{i\to j}(t)$ is the probability that node i is in the state S at time t when node j is fixed to the S state, $P_S^i(t)$ is the marginal probability that node i is in the state S at time t, and $\partial i\backslash j$ is the set of neighbors of node i excluding node j.

The above message-passing equations can be iterated in time, starting from initial conditions for cavity messages:

$$\theta^{i\to j}(0) = 1, \qquad (S9)$$

$$\phi^{i\to j}(0) = \delta_{q_i(0),I}, \qquad (S10)$$

where $q_i(t)$ is the state of node i at time t, $q_i(0)$ is the initial state of node i, and $\delta_{q_i(0),I} = 1$ if and only if $q_i(0) = I$. The marginal probabilities that node i is in a given state at time t are then given as

$$P_S^i(t+1) = P_S^i(0)\prod\nolimits_{k\in\partial i}\theta^{k\to i}(t+1), \qquad (S11)$$

$$P_R^i(t+1) = P_R^i(t) + \mu_i P_I^i(t), \qquad (S12)$$

$$P_I^i(t+1) = 1 - P_S^i(t+1) - P_R^i(t+1). \qquad (S13)$$

To infer the origin of an epidemic, we can run the DMP algorithm for each possible seed i by setting $P_S^i(0) = 0$, $\phi^{i\to j}(0) = 1$, $P_S^k(0) = 1$ for $k \neq i$, and $\phi^{k\to j}(0) = 0$ for $k \neq i$ in the initial condition. The joint probability of the observations is approximated using a mean-field-type approach as a product of the marginal probabilities provided by the dynamic message passing,

$$P(\mathcal{O}|i) \propto \prod_{\substack{k\in\mathcal{O}\\ q_k(t_o)=S}} P_S^k(t,i) \prod_{\substack{l\in\mathcal{O}\\ q_l(t_o)=I}} P_I^l(t,i) \prod_{\substack{m\in\mathcal{O}\\ q_m(t_o)=R}} P_R^m(t,i)\,, \qquad (S14)$$

where $\mathcal{O}$ is the set of observations, t_o is the observation time, and $P_S^k(t,i)$ is the marginal probability of the state S for node k at time t estimated using the DMP equations in which node i is the epidemic origin. The inferred epidemic origin is selected as the node that maximizes $P(\mathcal{O}|i)$.

Solving the DMP equations using iterations

The DMP algorithm has a competitive performance in inferring epidemic origin. However, it was not designed specifically for the problem of inferring unobserved infections. We modified the DMP algorithm to estimate the infection probability for each node in this study.

For the observation model used in this study, most observed individuals were only tested once given the low daily testing probability and the relative short simulation period. As a result, it is usually not possible to directly observe the activation or infection times for tested individuals. In addition, as only a small fraction of individuals' states are observed, solving the DMP equations with sparse observation is difficult. Here we propose an iteration approach to approximately solve the DMP equations.

First, we set the initial condition based on sparse observations. For each observed susceptible (S) individual k at time t_o, we set $P_S^k(0) = 1$, $\theta^{k\to j}(t) = 1$ and $\phi^{k\to j}(0) = 0$. As the SIR dynamics is irreversible, those individuals remain to be susceptible until the observation time t_o. To incorporate this information, during the iteration from time $t = 1$ to $t = T$, we forced $P_S^k(t) = 1$, $\theta^{k\to j}(t) = 1$ and $\phi^{k\to j}(t) = 0$ for $t = 1$ to t_o. For observed individuals with the states I and R, their initial states are uncertain. As a result, we assume they are all possibly infected at the beginning of the epidemic. For each observed infected (I) patient l at time t_o, we set $P_S^l(0) = 1 - 1/H$, $\theta^{k\to j}(0) = 1$ and $\phi^{k\to j}(0) = 1/H$ as the initial condition, where H is the total number of observed individuals with the state I or R. During the iteration, we forced $P_S^l(t) = 0$, $\theta^{l\to j}(t) = 0$ for $t = t_o$ to T and $\phi^{l\to j}(t_o) = 1$ (as node l is at the state I at time t_o). For each observed recovered (R) patient m at time t_o, we set $P_S^m(0) = 1 - 1/H$, $\theta^{m\to j}(0) = 1$ and $\phi^{m\to j}(0) = 1/H$ as the initial condition. During the iteration, we forced $P_S^m(t) = 0$, $\theta^{m\to j}(t) = 0$ and $\phi^{m\to j}(t) = 0$ for $t = t_o$ to T.

Second, to represent the uncertainty in the transmission probability λ_{ij}, we used two versions of the modified DMP algorithm. In the first, denoted as DMP1, transmission probabilities were set as a constant, the mean of a uniform distribution; in the second, denoted as DMP2, transmission probabilities for all individuals were randomly drawn from a uniform distribution, same as in the ensemble inference algorithm.

Using the modified DMP algorithm, we integrated the dynamic message passing equations from time $t = 1$ to T. The infection probability of individual i at time T was estimated using $P_I^i(T)$.

Discussion on the performance of the modified DMP algorithm

Previous studies showed that the DMP algorithm can support accurate inference of epidemic origin and epidemic parameters [3,4]. However, in this work, the performance of the modified DMP algorithm was not satisfactory for many networks, even for trees where the DMP equations are exact. To confirm that we have implemented the DMP algorithm properly, we reproduced the analysis in Ref. [3] (i.e., infer epidemic origin) in ER networks and found that the algorithm can identify the epidemic origin accurately.

There are two potential reasons for the degraded performance of the modified DMP algorithm in this study. First, the observation model used in this study is not compatible with the one used in the DMP studies. In Ref. [3], the activation times of nodes were observed. The DMP equations were parameterized using information of activation times to capture the local dependency of activation times among neighbors. In this study, we observed the states of a small fraction of nodes at different times, which provide limited information on activation times. Second, solving the DMP equations given a small number of inputs is challenging. In our implementation, we iterated the DMP equations over time while fixing cavity messages for observed nodes. We found that after a few iterations, the cavity messages in the DMP equations tend to converge to the leading eigenvector of the system. A better approximation method may be used to improve the performance of the modified DMP algorithm.

Statistics of real-world networks

Basic statistics of the real-world networks used in experiments are provided in Table B.

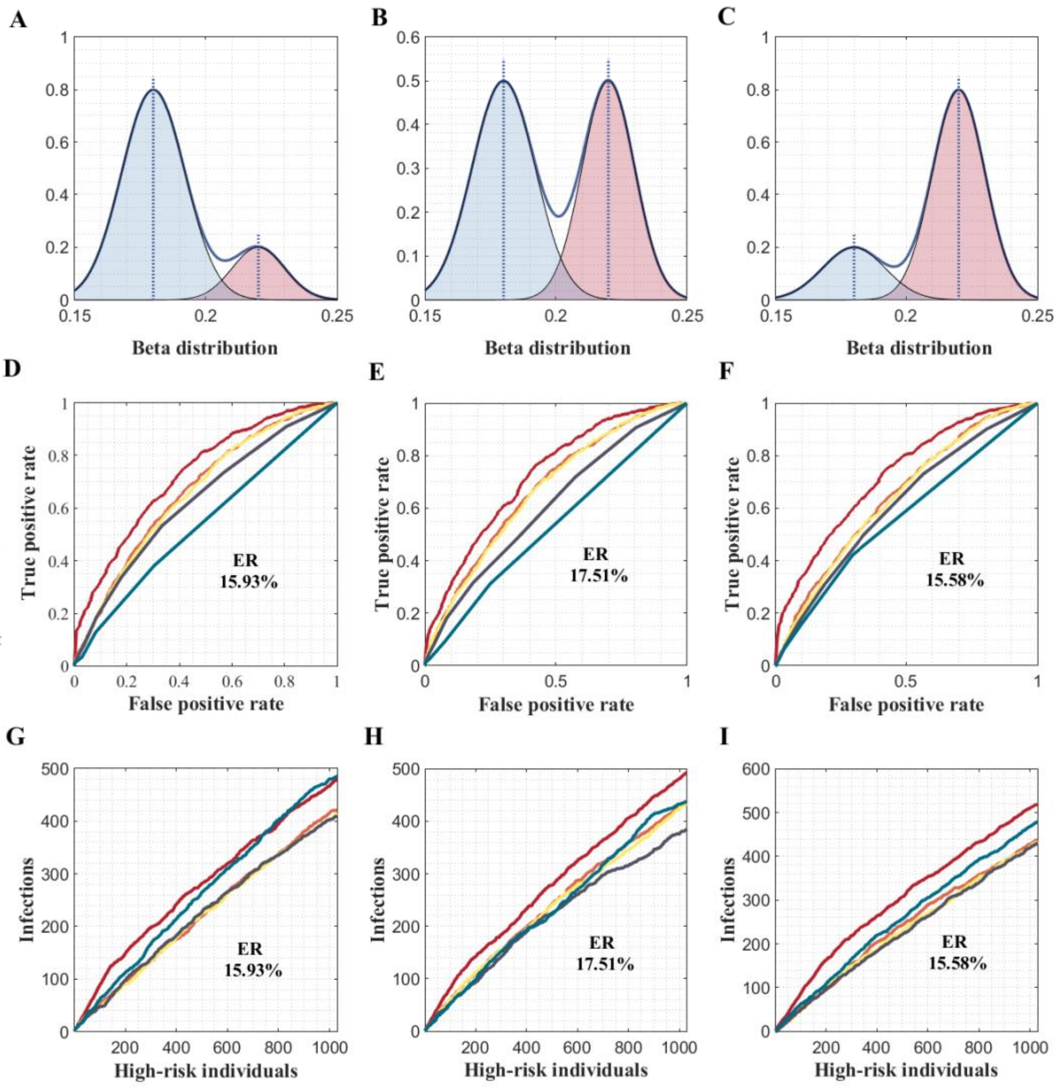

Fig A. Performance under different bimodal distributions of transmission rates. (A-C) Illustrations of three bimodal distributions of individual transmission rate. We used two Gaussian distributions to produce the bimodal distribution. (D-F) The ROC curves for various distributions of transmission rates are shown. Experiments were performed in an ER network with 3,000 nodes and a mean degree 4. (G-I) The numbers of infections identified among high-risk individuals selected by different methods.

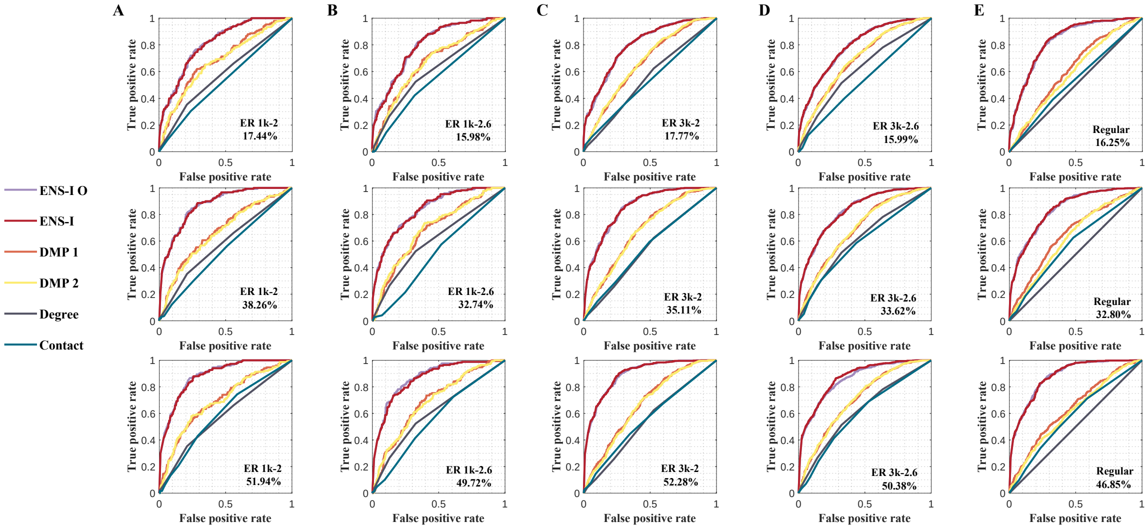

Fig B. ROC curves for different methods in random networks. The legend shows the network model, size of network, average degree, and the percentage of observed individuals. For instance, "ER 1k-2 17.44%" means an ER random network with $N = 1{,}000$ nodes and an average degree $\langle k \rangle = 2$, with 17.44% observed nodes. We perform experiments on ER random networks with (A) $N = 1{,}000$, $\langle k \rangle = 2$, (B) $N = 1{,}000$, $\langle k \rangle = 2.6$, (C) $N = 3{,}000$, $\langle k \rangle = 2$, (D) $N = 3{,}000$, $\langle k \rangle = 2.6$, and a random regular network with degree 4 (E). We compare six different methods: the original full version of the ensemble inference that considers interdependency of observations (ENS-I O), the expedited ensemble inference (ENS-I), modified dynamic message-passing with a fixed transmission rate (DMP1), modified dynamic message-passing with uniformly distributed transmission rates (DMP2), number of connections (Degree), and contact with observed infections (Contact).

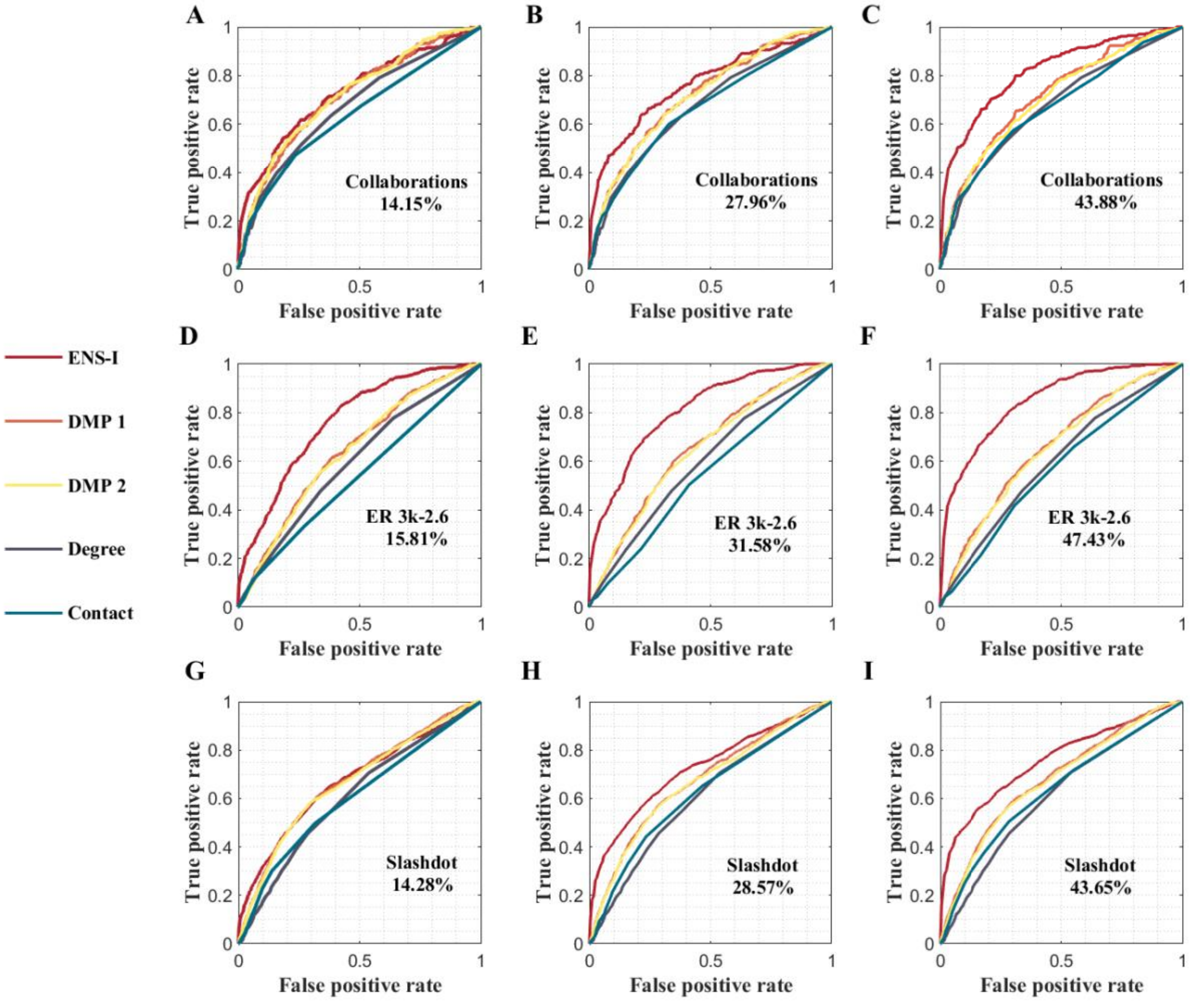

Fig C. Experiments using a power-law distribution of individual transmission rate. We perform experiments on three different networks and randomly draw individual transmission rate from a power-law distribution $P(\beta) \propto \beta^{-2.5}$. We compare five different methods: the expedited ensemble inference (ENS-I), modified dynamic message-passing with a fixed transmission rate (DMP1), modified dynamic message-passing with uniformly distributed transmission rates (DMP2), number of connections (Degree), and contact with observed infections (Contact).

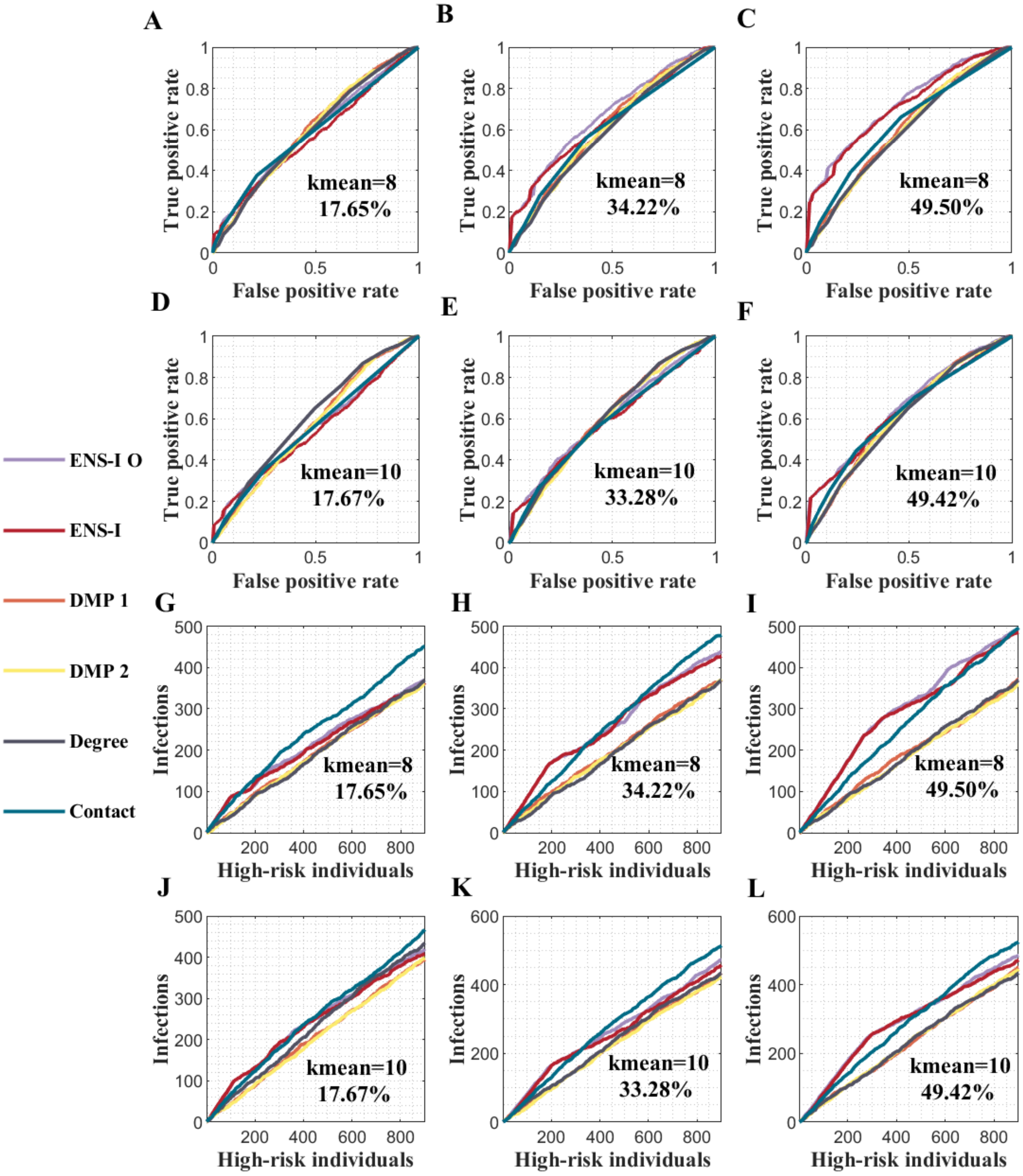

Fig D. Experiments in ER random networks with higher average degrees. (A-C) The ROC curves for an ER random network with 3,000 nodes and an average degree 8. (D-F) The ROC curves for an ER random network with 3,000 nodes and an average degree 10. (G-I) The numbers of infections identified among high-risk individuals selected by different methods in an ER network with an average degree 8. (J-L) The numbers of infections identified among high-risk individuals selected by different methods in an ER network with an average degree 10.

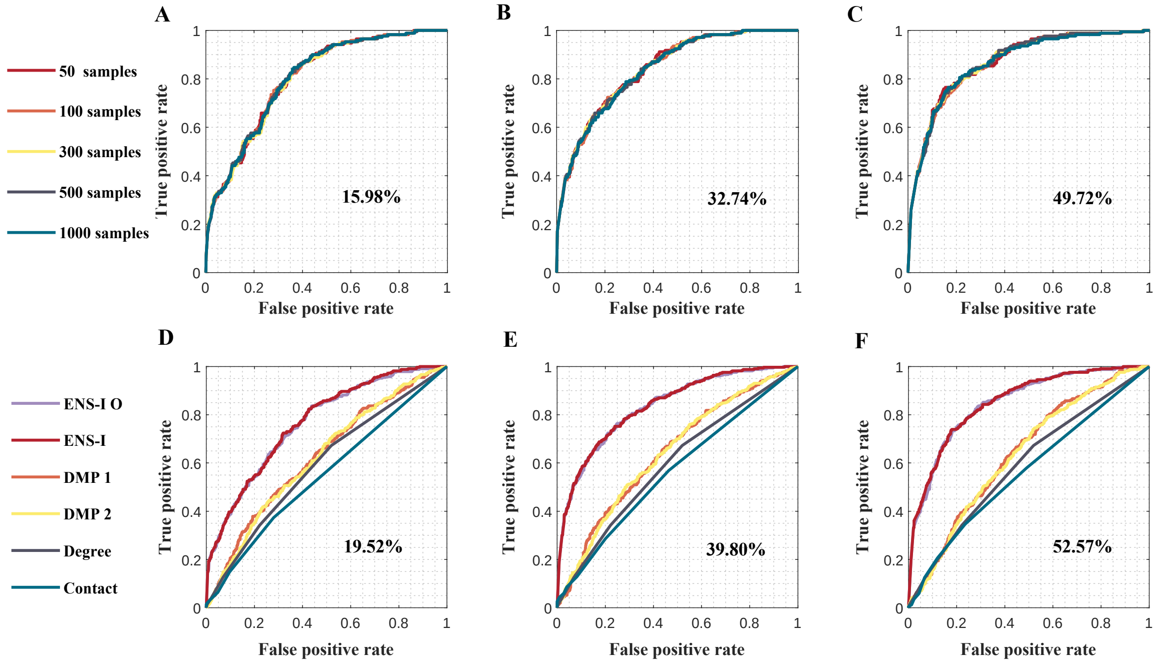

Fig E. Experiments with different numbers of ensemble members and using a contact tracing observation model. Experiments were performed in an ER network with 1,000 nodes and an average degree of 2.6. (A-C) Comparison of the ROC curves of the ensemble inference algorithm with different numbers of ensemble members. The percentage numbers show the percentage of nodes in the network that were observed. (D-F) Comparison of different methods using a observation model based on contact tracing.

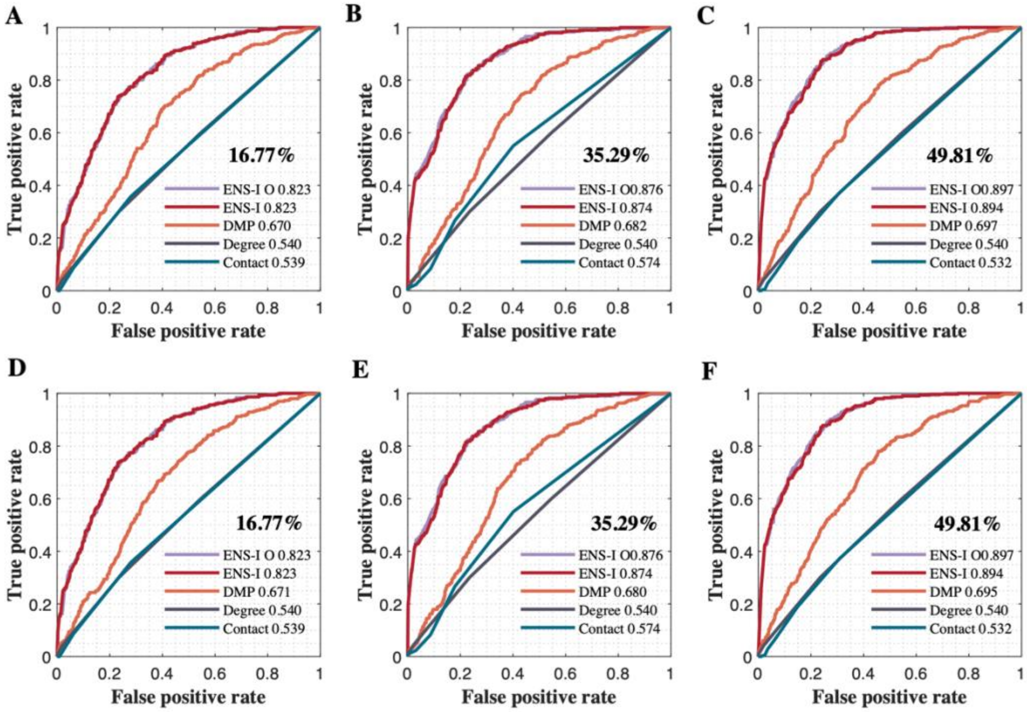

Fig F. The modified DMP algorithm with more accurate transmission rates. On an ER random network with $N = 1598$ nodes and an average degree of 2.4, we generated a group of transmission rates according to a bimodal distribution and simulated an outbreak for one week. About 15%, 30% and 45% of all nodes were observed. We used accurate transmission rates for each node in the DMP method in (A-C) and transmission rates with a 10% random noise in (D-E). For the modified DMP algorithm, more accurate transmission rates do not significantly improve the accuracy of inference.

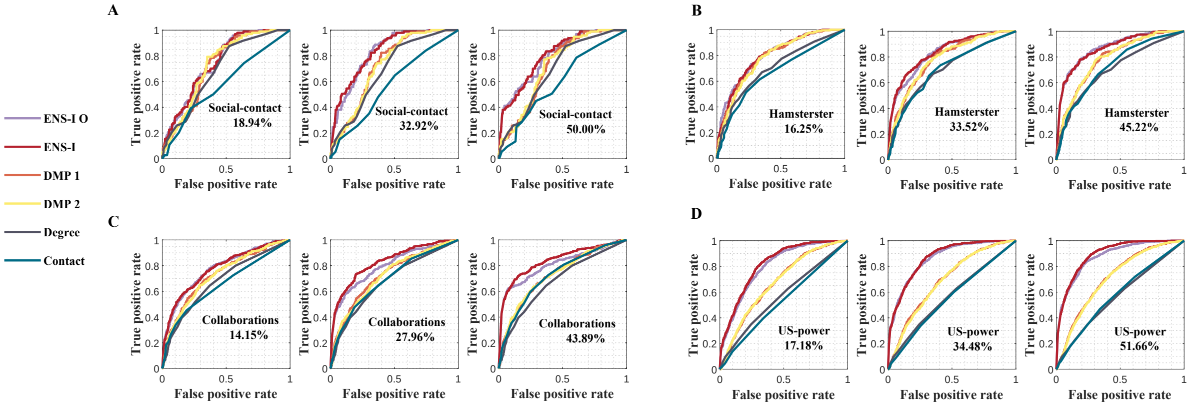

Fig G. Experiments in real-world networks with varying observation rates. We show results for networks with less than 5,000 nodes. We compare six different methods: the original full version of the ensemble inference that considers interdependency of observations (ENS-I O), the expedited ensemble inference (ENS-I), modified dynamic message-passing with a fixed transmission rate (DMP1), modified dynamic message-passing with uniformly distributed transmission rates (DMP2), number of connections (Degree), and contact with observed infections (Contact).

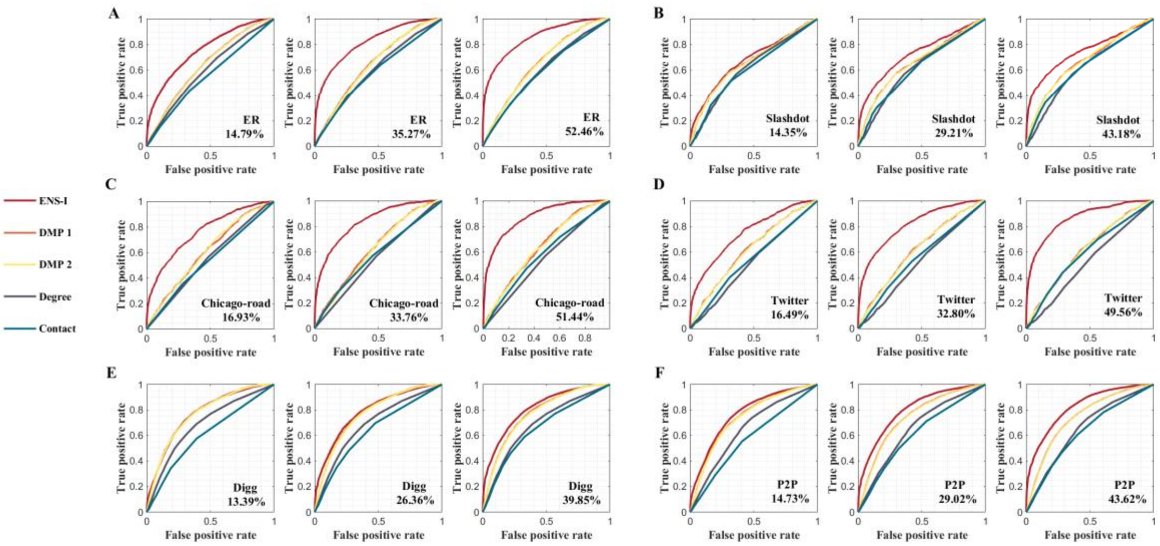

Fig H. Additional experiments in networks with varying observation rates. We show results for networks with more than 5,000 nodes. We compare five different methods: the expedited ensemble inference (ENS-I), modified dynamic message-passing with a fixed transmission rate (DMP1), modified dynamic message-passing with uniformly distributed transmission rates (DMP2), number of connections (Degree), and contact with observed infections (Contact). The original full version of the ensemble inference is computationally expensive for large-scale networks. As a result, we did not run the original algorithm for these networks.

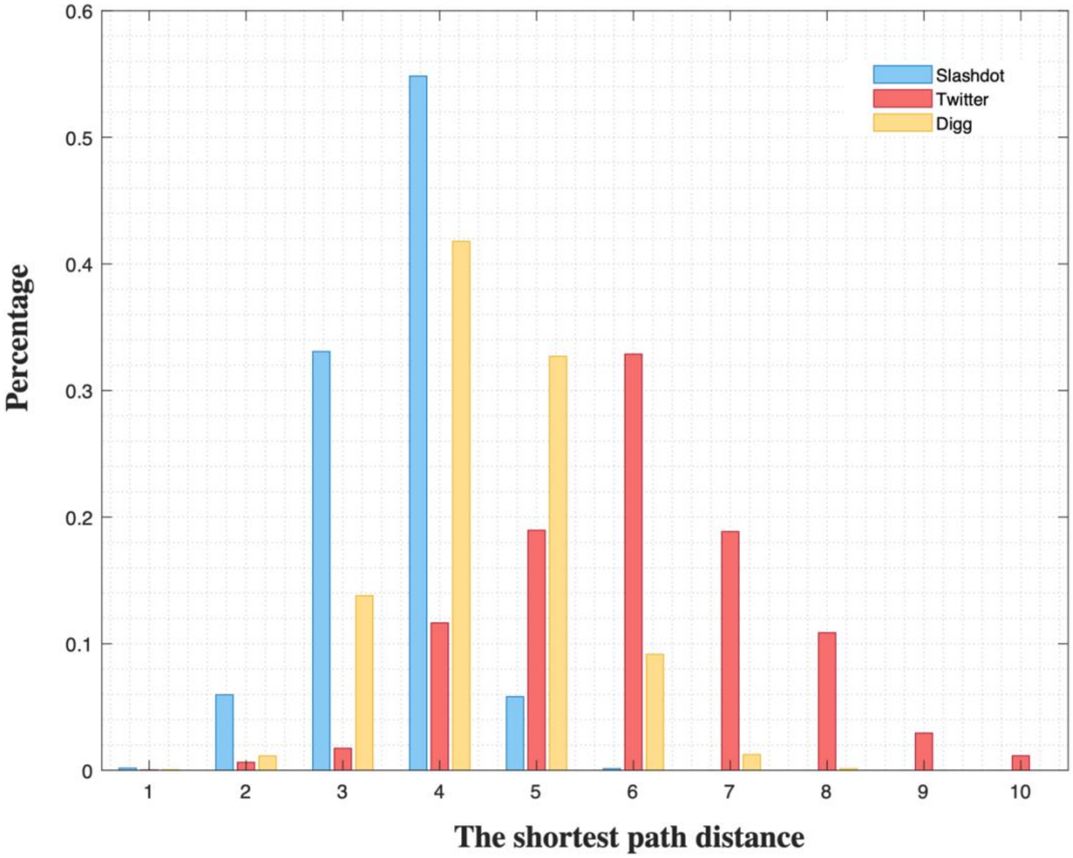

Fig I. Distributions of the shortest distance between pairs of observed nodes in three networks. We performed the analysis for three real-world networks (Slashdot, Digg, and Twitter). For each network, around 15% nodes were observed.

Table A. Configurations of epidemic models and inference. The first column shows the networks used in experiments. Transmission rate distribution is the bimodal distribution of β in outbreak simulations, produced by superimposition of two Gaussian distributions ($\mathcal{N}(\mu, \sigma^2)$ is a Gaussian distribution with the mean μ and the variance σ^2). Prior transmission rate is the uniform distribution used to sample the individual transmission rates in the ensemble inference. Daily observation rate is the probability of each node in the state S, I or R to be observed on each day. Total observation shows the percentage of observed individuals in the network. The last column shows the corresponding figure in the main text.

Network	Transmission rate distribution	Prior transmission rate	Daily testing probability [S/I/R]	Total observation	Figure
ER1k-2.6	$\mathcal{N}(0.18,0.012)$, $\mathcal{N}(0.22,0.01)$	[0.15,0.25]	[0.011,0.11,0.014]	15.98%	Fig 3A
			[0.023,0.23,0.028]	32.74%	Fig 3B
			[0.034,0.34,0.043]	49.72%	Fig 3C
Social contact network	$\mathcal{N}(0.052,0.004)$, $\mathcal{N}(0.065,0.003)$	[0.04,0.075]	[0.011,0.11,0.014]	18.94%	Fig 5A
Hamsterster				16.25%	Fig 5B
Collaborations				14.15%	Fig 5C
US-power	$\mathcal{N}(0.18,0.012)$, $\mathcal{N}(0.22,0.01)$	[0.15,0.25]		17.18%	Fig 5D
Chicago-road	$\mathcal{N}(0.052,0.004)$, $\mathcal{N}(0.065,0.003)$	[0.04,0.075]	[0.0064,0.064,0.0071]	16.93%	Fig 5E
Slashdot				14.35%	Fig 5F
Digg				13.39%	Fig 5G
Twitter				16.49%	Fig 5H
P2P				14.73%	Fig 5I

Table B. Basic statistics of the real-world networks used in experiments.

Network	Network Description	Node	Edge	Mean degree	Maximum degree
Social contact network	Social contact network for Science Gallery visitors on 16th July	322	1254	9.161	6
Hamsterster	Friendship of Hamsterster households	1576	4032	5.117	147
Collaborations	Scientific collaborations among institutions in New Zealand	1511	4273	5.66	551
US-power	Power grid of the Western States of the USA	4941	6594	2.669	19
Chicago-road	Road transportation network of the Chicago	12982	39018	6.011	14
Slashdot	A network of friends for sharing news	11227	20908	5.146	414
Digg	Reply network of the social news website Digg	30398	87627	5.765	310
Twitter	Twitter user–user following information	23370	33101	2.832	239
P2P	A network of Gnutella hosts from 2002	62586	147892	4.726	95

Supplementary References

1. Melnik S, Hackett A, Porter MA, Mucha PJ, Gleeson JP. The unreasonable effectiveness of tree-based theory for networks with clustering. Phys Rev E. 2011;83: 036112. doi:10.1103/PhysRevE.83.036112

2. Anderson JL. An Ensemble Adjustment Kalman Filter for Data Assimilation. Mon Wea Rev. 2001;129: 2884–2903. doi:10.1175/1520-0493(2001)129<2884:AEAKFF>2.0.CO;2

3. Lokhov AY, Mézard M, Ohta H, Zdeborová L. Inferring the origin of an epidemic with a dynamic message-passing algorithm. Phys Rev E. 2014;90: 012801. doi:10.1103/PhysRevE.90.012801

4. Lokhov A. Reconstructing Parameters of Spreading Models from Partial Observations. Advances in Neural Information Processing Systems. 2016. pp. 3467–3475. Available: https://proceedings.neurips.cc/paper/2016/hash/404dcc91b2aeaa7caa47487d1483e48a-Abstract.html
