# Supplementary material for: Ensemble inference of unobserved infections in networks using partial observations
Source: PLoS Comput Biol. 2023 Aug 7;19(8):e1011355. doi: 10.1371/journal.pcbi.1011355 (PMC10434926; doi:10.1371/journal.pcbi.1011355)
Supplement: Attachment — Submitted filename: Response.docx [file pcbi.1011355.s002.docx]

Reviewer #1: In this study, the authors proposed an ensemble inference method to infer unobserved infections under the condition of unknown model parameters. This method was applied to an SIR model in networks and was validated in both synthetic and real-world networks. The performance of the inference method was shown to be better than several competing methods. The authors also examined the computational complexity of the algorithm, showing its potential for applications in large-scale networks. This study addressed an important challenge in epidemic inference in real-world settings – uncertainty in model parameters. This work makes a meaningful contribution to the literature on inference problems of epidemic processes in networks, with potential implications in real-world applications. However, I have several suggestions before accepting the manuscript.

Response: We appreciate the reviewer’s positive feedbacks on this study.

1. In “Experiments on simulated networks”, numerical simulations were done only in ER and regular networks. Similar experiments should be performed in model-generated scale-free networks.

Response: In the revised manuscript, we have included the experiment on scale-free networks. The SF network was generated using the configuration model, with a power-law degree distributions $P\left( k \right)\sim k^{-\gamma}$, where the power-law exponent $\gamma=2.5$. The ensemble inference algorithm had a better performance than other methods. Please find changes in lines 346-349 and Figure 4 in the revised manuscript.

2. To better understand the algorithm, I suggest to move the “Pseudo-code and implementation” section in the SI to the section “Materials and methods” in the main text. Readers need more details on the algorithm implementation.

Response: Great suggestion. We have moved the pseudo-code to the Materials and methods section. Please see line 225 in the revised manuscript.

3. In the discussion section, it would be helpful to list a few application settings where the proposed algorithm can be used.

Response: A discussion on the potential applications of the inference algorithm has been added to the Discussion section. The proposed inference method can be applied in several settings. For instance, during an outbreak of an emerging pathogen with a high rate of asymptomatic infection, partially observed infections coupled with detailed contact information can be used to support the inference of unobserved spreaders. In hospital settings, the inference method can be used to identify asymptomatic carriers of antimicrobial-resistant organisms based on sparse observations of clinical infections, limiting the nosocomial transmission of these pathogens. Please see changes in lines 390-395 in the revised manuscript.

4. The format of references should be checked carefully, such as [1], [36]

Response: We have fixed the format issues in the references.

Above all, it is a novel paper. I would suggest acceptance with a minor revision.

Reviewer #2: Summary:

The paper presents an ensemble Bayesian inference method to infer missing or unreported infections in networks using partial observations. The objective is to estimate the probability of unobserved infections and inform prospective interventions to control outbreaks. The proposed method is able to handle uncertainty in model structure and demonstrates robust predictions in infectious disease forecasting. The algorithm is evaluated on both synthetic and real-world networks and outperforms considered baselines.

The paper uses a network-based SIR model with individual transmission rates drawn from a bimodal distribution and a common recovery rate for infected individuals. To model the real-world observational process, individuals are tested with a probability on each day depending on their states. The ensemble Bayesian inference algorithm uses these partial observations to estimate the probability to estimate the probability of individuals being in a particular state (S, I, R) on each day of an outbreak, given partial observations. For each (node, state, time) tuple, the probability of the node being in that particular state at that time is the probability of interest. It is computed using master equations that account for network structure, time dependencies, and transmission & recovery rates.

The key novel contribution here is that the authors account for uncertainty in transmissibility rates using an ensemble of possible realizations of these probabilities and update them sequentially using the sparse observations, backward temporal propagation, cross-ensemble covariability adjustment, and model integration. Each member of the ensemble differs in the network edge probabilities, which are chosen randomly from a bimodal distribution. Throughout, 100 ensemble members were used in the inference.

The performance of the ensemble inference algorithm is compared to several baselines, including degree, contact, and modified dynamic message-passing approach (DMP1 and DMP2), which ranked individuals' infection probability. For the experiments, both synthetic and publicly available real-world networks have been used. Networks have been generated using the Erdős–Rényi (ER) model for the synthetic network experiments. The ensemble inference algorithm outperforms other methods, even when the distribution of transmission rate has been mis-specified. The accuracy of the ensemble inference algorithm increases with more observations, but the marginal benefit diminishes with increasing observations. For the inference on real-world networks, performance for the top-ranked (high-risk) individuals has been considered. The ensemble inference algorithm identifies more infections than other methods for a given number of estimated high-risk individuals. Additional experiments have been conducted to establish the effectiveness of the ensemble inference algorithm in homogeneous networks, including ER networks with a larger number of nodes, networks with transmission rate drawn from a power-law distribution, and random regular graphs.

Response: We appreciate the reviewer’s comprehensive summary and constructive feedbacks. In the revised manuscript, we performed extensive additional experiments according to the reviewer’s suggestions. A list of 18 major changes or experiments is shown below.

1. Provided a formal problem statement in the main text.
2. Included additional literature in the introduction.
3. Separated the epidemic model and observation model, discussed the role of observation model in inference.
4. Moved the master equations and the pseudo-code to the main text.
5. Updated Figure 1 and its caption.
6. Evaluated performance for tree structure.
7. Evaluated performance in denser ER random networks.
8. Evaluated performance for different numbers of ensemble members, provided a detailed discussion on ensemble size.
9. Reproduced the original DMP algorithm to infer epidemic origin and confirmed that the algorithm was properly implemented.
10. Evaluated the modified DMP algorithm given more accurate parameters, provided a discussion on the performance of the modified DMP algorithm in this study.
11. Evaluated performance for contact tracing.
12. Evaluated performance for two other bimodal distributions of transmission rates.
13. Evaluated performance for scale-free networks.
14. Updated several real-world networks in experiments.
15. Performed additional experiments on computational complexity with varying ensemble size and average degree.
16. Clarified the difference between the full inference algorithm and the simplified algorithm.
17. Examined the distance between observed nodes in three real-world networks.
18. Restructured the discussion section by removing redundant information and adding references.

A point-by-point response to reviewer’s questions is provided below.

Major comments:

Problem is not formally stated: The basic objective is to infer unobserved infections. The setup is provided in the "Inference" subsection in "Material and Methods". However, there is no formal problem statement where the set of observed infections is specified. It has to be understood from the section on epidemic model. Also, the observation model should be separated from the epidemic model as the method used for observation clearly can be applied to multiple epidemic models.

Response: Thanks for this suggestion. We agree the inference problem was not formally introduced in the previous manuscript. In the revised manuscript, we have added a subsection “Problem statement” after the introduction of epidemic and observation models. Please see changes in lines 158-166 in the revised manuscript. In addition, we have separated the observation model from epidemic models and created a new subsection “Observation model”. See lines 142-156 in the revised manuscript.

Number of configurations in the ensemble: The main novelty of this paper is
in the use of ensembles of configurations. Throughout, 100 members are used in the ensemble. Even if we pick from a small finite set of edge probabilities, for a moderate-sized network, the number of possible combinations of transmission probabilities is very large. The authors do not provide an explanation for why only 100 members are considered. The reviewer is of the opinion that 100 configurations might not be representative. Considering that this is a key idea in the paper, more experimental evaluation needs to done.

Response: This is a very good question. To address this question, we first performed an experiment evaluating how ensemble size would impact the inference performance. Specifically, we ran the ensemble inference algorithm using 50, 100, 300, 500, and 1000 ensemble members and found that the performance remained similar even if more ensemble members were used (see lines 312-315 in the revised manuscript and S5A-C Figure in the revised supplementary material). Additionally, we have added an in-depth discussion on the potential reasons for this phenomenon.

Indeed, given the high dimensionality of the epidemic model, it is surprising that a few hundred ensemble members in the inference algorithm can yield a good performance. This could be due to several potential reasons. First, the purpose of using an ensemble of transmission rates for each edge is to avoid the large errors caused by using a single wrong parameter. As long as the ensemble can cover a reasonable range of edge-specific transmission rates, estimation errors will likely be limited and damped. In other words, the ensemble does not need to be a representative sample of all possible combinations of edge-specific transmission rates. Second, the ensemble of model states $P(X_{i}^{t})$ for many individuals can be dynamically adjusted using covariablity with their neighbors. This “nudge” procedure can provide partial correction of model states so that the model can better fit observations. This phenomenon was also observed in the comparison of particle filter (or sequential Monte Carlo) [1] and ensemble Kalman filter [2,3]. In particle filter, the distribution of model states is represented by a weighted average of a large number of particles, each representing a potential combination of model parameters and variable. During model update, the weights of particles are adjusted but the model states remain the same (unless particles degenerate, i.e., dominated by a small number of particles, and needs to be re-sampled). In this case, it was found that the number of required particles grows exponentially with the number of parameters and variables [4], which limits its application to high-dimensional systems. In contrast, the ensemble Kalman filter, although suboptimal for nonlinear models, can be applied to high-dimensional systems such as weather models [2] and metapopulation epidemic models [5] using a relatively small number of ensemble members. The key reason is that ensemble members can be nudged according to observations, which creates more flexibility in model states. More studies are needed to explore the impact of ensemble size on inference performance. Please find changes in lines 415-437 in the revised manuscript.

No theoretical guarantees: Theoretical treatment could have greatly strengthened this paper. Some results, even under the assumption of some restricted family of graphs, inference model, and model parameters could throw light on the ability and limitations of the proposed method. For example, in line with Lokhov et al. (2014), the authors could have attempted some result for trees: How does the ensemble-based algorithm perform on trees for which the master equations are accurate, and for which the DMP algorithm provides exact inference.

Response: We appreciate the suggestion for providing theoretical guarantees. Indeed, the master equations are accurate for trees; however, obtaining a theoretical guarantee for ensemble methods is not trivial. For instance, the ensemble Kalman filter was developed in 1990s but the theoretical proofs of convergence under different conditions were recent [6–8]. We therefore will focus on the practical performance of the inference algorithm in this study. In the revised manuscript, we have included an experiment on trees. The ensemble inference algorithm performed better than other methods (Fig. 2). In particular, the accuracy of top-ranking individuals was high, possibly due to the accurate master equations in tree structure. Please see changes in lines 260-272 in the revised manuscript.

Literature survey: The authors have missed several publications on missing infections. This is an important topic that has been very well studied in epidemiology, computer science, and physics. Here are some examples.

Rozenshtein, P.; Gionis, A.; Prakash, B. A.; and Vreeken, J. 2016. Reconstructing an Epidemic Over Time. In Proceedings of the 22nd ACM SIGKDD International Conference on Knowledge Discovery and Data Mining, KDD ’16, 1835–1844. New York, NY, USA: Association for Computing Machinery. ISBN 9781450342322.

Xiao, H.; Aslay, C.; and Gionis, A. 2018. Robust Cascade Reconstruction by Steiner Tree Sampling. In 2018 IEEE International Conference on Data Mining (ICDM), 637–646.

Jang, H.; Pai, S.; Adhikari, B.; and Pemmaraju, S. V. 2021. Risk-aware Temporal Cascade Reconstruction to Detect Asymptomatic Cases : For the CDC MInD Healthcare Network. In 2021 IEEE International Conference on Data Mining (ICDM), 240–249.

Ritwick Mishra, Jack Heavey, Gursharn Kaur, Abhijin Adiga, Anil Vullikanti, Reconstructing an Epidemic Outbreak Using Steiner Connectivity, AAAI'23.

Response: We thank the reviewer for pointing us to those relevant works. In the revised manuscript, we have included more literature in computer science and physics, focusing on two lines of research – likelihood maximization and Steiner tree sampling. Please find changes in lines 83-93 in the revised manuscript.

The following publication is also quite relevant.

Lokhov, Andrey. "Reconstructing parameters of spreading models from partial observations." Advances in Neural Information Processing Systems 29 (2016).

It uses a DMP-based inference algorithm to estimate model parameters from partial observations. The reviewer suggests that the authors use this work in the following manner to obtain a baseline: (1) Run this algorithm to obtain estimates of the model parameters and then (2) use these model parameters along with the standard DMP algorithm to obtain estimates for probability that a node is infected. Xiao et al. (2018) consider robust cascade reconstruction and have a similar framework as this paper.

Response: This is a good suggestion. We attempted to estimate model parameters using the method in Lokhov 2016. However, inference of model parameters needs partial information on the activation times of nodes. Observing activation times of nodes is difficult for the observation model used in this study and is in general challenging in real world due to the uncertain time period from infection to a positive test. To circumvent this difficulty, we assigned the actual transmission rates (and the actual transmission rates with a 10% random Gaussian noise) to the modified DMP algorithm. The performance of the modified DMP algorithm was still not satisfactory (S6 Figure), likely due to the observation model and issues solving the DMP equations using sparse observations.

We have included a detailed discussion on the performance of the modified DMP algorithm in this study. To confirm that we have implemented the DMP algorithm properly, we reproduced the analysis in Ref. [9] (i.e., infer epidemic origin) in ER networks and found that the algorithm can identify the epidemic origin accurately. There are two potential reasons for the degraded performance of the modified DMP algorithm in this study. First, the observation model used in this study is not compatible with the one used in the DMP studies. In Ref. [9], the activation times of nodes were observed. The DMP equations were parameterized using information of activation times to capture the local dependency of activation times among neighbors. In this study, we observed the states of a small fraction of nodes at different times, which provide limited information on activation times. Second, solving the DMP equations given a small number of inputs is challenging. In our implementation, we iterated the DMP equations over time while fixing cavity messages for observed nodes. We found that after a few iterations, the cavity messages in the DMP equations tend to converge to the leading eigenvector of the system. A better approximation method may be used to improve the performance of the modified DMP algorithm. Please find changes in the revised supplementary materials.

Secondly, the baselines degree and contact with observed infections have used as baselines in many of the above-mentioned works. It is not surprising that the proposed algorithm does better than them.

Response: We agree degree and contact were widely used in previous studies. However, as these strategies are widely used and are easy to implement in real-world settings, we decide to include them in experiments. We have highlighted this motivation

A very specific observation model: Many inference models have been considered in the literature. The authors consider a random testing based inference model and their entire study is based on this model. This is important as the complexity and the nature of the problem changes with the observation model.

Response: We thank the reviewer for this insightful comment. In the revised manuscript, we have included a discussion on observation models (see lines 439-449 in the revised manuscript).

Observation model is key to inference problems. The computational complexity and the nature of the inference problem change with the observation model. In fact, in many studies, observation model often determines the specific technical approach to solve problems. Several observation models were used in literature, including the infection times of all or partial nodes and the states of all or partial nodes at a given snapshot. In this study, we used a daily testing strategy to mimic tests among partial populations performed at various time points, which agrees with most real-world testing scenarios. We note that the inputs of the ensemble inference algorithm are the states of a fraction of population observed at different times (i.e., $\mathcal{D}=\{(i_{o},t_{o},O)\}$). As long as an observation model can provide such information, the ensemble inference algorithm is applicable. This offers the flexibility of the inference algorithm to work for different observation models.

Discussion section could be strengthened: The first two paragraphs are merely summary that has already been covered in other parts of the paper. The third paragraph could benefit from references. The fourth paragraph needs more careful analysis. Since this is an experimental work, unless similar analysis is made for models such as SIS or SI, the authors cannot claim generality. If the authors had proven some rigorous theoretical guarantees, then may be they could have been extended to other models. It can be stated as future work though.

Response: Great suggestions. We have removed the first two and the fourth paragraphs from the discussion following the reviewer’s suggestions. In addition, we have included more references in the third paragraph. We further added discussions on the application, ensemble members, and observation model in the Discussion section. See lines 390-449 in the revised manuscript.

Networks: Some of the real-world networks are not representative in the context of epidemiology. Epidemiological applications include spread of diseases, rumor spread, or spread of malware. The authors evaluate their algorithms on transport and protein networks. Why were these networks chosen? No networks correspond to infectious disease epidemiology. There are networks on human interactions that are publicly available and have been applied for disease spread studies. See for example:

SocioPatterns: Cattuto, Ciro, et al. "Dynamics of person-to-person
interactions from distributed RFID sensor networks." PloS one 5.7 (2010):
e11596.

Response: We totally agree with the reviewer. In the revised manuscript, we have removed biological networks and replaced them with a face-to-face proximity network (<http://www.sociopatterns.org/datasets/infectious-sociopatterns/>), an online social network (<http://konect.cc/networks/petster-hamster-household/>), and a scientific collaboration network (<https://networks.skewed.de/net/new_zealand_collab>). Please see changes in Fig. 5 and Fig. 6.

Also, the average degree of ER graphs is quite low. More experiments with denser graphs could strengthen the paper.

Response: Agree. We have performed experiments in denser ER networks with a mean degree of 8 and 10. The performance of the ensemble inference algorithm degraded for loopy networks, likely due to the lower accuracy of the master equations in denser networks. But the ensemble inference algorithm can still identify more infections in the top 10% higher-risk nodes, especially with more observations. Future works are needed to improve the performance for loopy network structure. Please see changes in lines 309-312 in the revised manuscript and S4 Figure.

Computational complexity: An analysis of the computational complexity is presented with varying network size and number of observations. The analysis was conducted on ER graphs and is quite detailed. But it is not clear what the size of network means. A network's size depends not only on the number of nodes but also edges. This information is not provided in Figure 5. The complexity will also depend on the number of ensembles. This is not addressed.

Response: This is a good point. In the previous manuscript, network size means the number of nodes with a fixed average degree. We have clarified the definition in the revised manuscript. Additionally, we have performed experiments on the computational time for changing average degree of networks and different number of ensemble members. Please see details in lines 372-386 and Fig. 7 in the revised manuscript.

Bimodal distribution: The authors state that the reason for choosing bimodal distribution is to mimic the situation where a subpopulation is partially protected. But they seem to have considered the specific case of 50% of the population (on an average) being protected. It would be interesting to know how the performance changes by varying the parameters of the bimodal distribution and how probabilities are assigned.

Response: Thanks for this suggestion. We have performed experiments for two other bimodal distributions (20% nodes with lower transmission rates versus 80% nodes with lower transmission rates). The ensemble inference algorithm outperformed other methods for both scenarios. Details can be found in lines 331-338 in the revised manuscript and S1 Figure.

Expedited integration of master equations: What is the difference between ENS-I O and ENS-I? In the expedited ensemble inference (ENS-I), the ensembles are being processed independently. Does this have something to do with cross-ensemble covariance?

Response: We have clarified the different between the two methods. The major difference is the computational time. In the full inference algorithm ENS-I O, to compute the likelihood $P\left( X_{i_{o}}^{t_{i_{o}}}=O | X_{i_{o}}^{t} \right)$, we need to run the master equations for each observation $X_{i_{o}}^{t}$. Specifically, for each observation $X_{i_{o}}^{t}$, we set $P\left( X_{i_{o}}^{t}=S \right)=1$ at time $t$ while keeping the states of other nodes unchanged, run the master equations till time $t_{i_{o}}$, and obtain $P\left( X_{i_{o}}^{t_{i_{o}}}=O | X_{i_{o}}^{t}=S \right)$. We need to additionally run master equations for $P\left( X_{i_{o}}^{t}=I \right)=1$ and $P\left( X_{i_{o}}^{t}=R \right)=1$ to get the posterior distribution. If there are $N_{o}$ observations, we need to run the master equations for $3N_{o}$ times. In contrast, in the simplified algorithm ENS-I, we want to compute $P\left( X_{i_{o}}^{t_{i_{o}}}=O | X_{i_{o}}^{t} \right)$ for all observed individuals simultanesously. To do this, at time $t$, we set $P\left( X_{i_{o}}^{t}=S \right)=1$ for all observed nodes $i_{o}$, run the master equations, and obtain $P\left( X_{i_{o}}^{t_{i_{o}}}=O | X_{i_{o}}^{t}=S \right)$ for all $i_{o}$. Following the same method, we can compute $P\left( X_{i_{o}}^{t_{i_{o}}}=O | X_{i_{o}}^{t}=I \right)$ and $P\left( X_{i_{o}}^{t_{i_{o}}}=O | X_{i_{o}}^{t}=R \right)$ for all $i_{o}$. As a result, we only need to run the master equations for 3 times for all $N_{o}$ observations. The assumption for the simplified algorithm ENS-I is that the states of observations are relatively independent, so changing the states of other observed nodes won’t impact much of the states of the focal node. Given the sparse observation and the distance between observed nodes, this assumption is reasonable. We have added a discussion in the revised supplementary materials.

Speed-up: In the supplement, the following statement is made: For randomly observed sparse observations, we can assume the interdependency of their states is negligible and estimate the posterior of i_o ... While this speeds up computation of the likelihoods, it is not clear if this independence can be assumed for all scenarios. Firstly, synthetic graphs (on which experiments were conducted) like ER-graphs have low clustering coefficient and other "high-girth" properties. Combined with the random observations model used in this paper, it is possible that there is some minimum distance between observed nodes. This could be the reason for the good performance. More analysis in this regard will help as this would help in scaling up to large graphs. Secondly, it is not clear if this works with other more realistic observation models like contact-tracing, where observed nodes are within a constant number of hops from each other.

Response: Agree it is important to check the assumption of independence among observed individuals. To check the validity of the relative independency of observations, we performed an analysis to examine the distance between observed individuals in the contact network. We computed the distribution of the pairwise shortest distance between observed nodes in three real-world networks – Slashdot, Twitter, and Digg (S9 Figure). On average, the distance between most pairs of observations is larger than 2 steps. For a small transmission rate, we can assume the observed nodes are relatively independent. This analysis is reported in the revised supplementary materials.

Regarding the observation model, we further tested contact tracing in ER random networks. In this model, each node was tested with a daily testing probability, similar to the previous observation model. However, once an individual was tested positive on day $t$, all neighbors of infection in the contact network were tested on the next day $t+1$. Contact tracing can discover infections among the neighbors of infected individuals. Therefore, observed infections are not independent from each other. Nevertheless, the ensemble inference algorithm still achieved a satisfactory performance (S5D-F Figure). We have included this analysis in lines 340-344 in the revised manuscript.

Minor:

Fig. 1c: It does not contain enough information about the concepts that the authors are trying to illustrate. It can be greatly improved. What are the blue and pink colors?

Response: We have updated Figure 1 in the main text. Specifically, we have added representations of ensemble distributions of model states and transmission rates and equations for model updates. We use red color to represent information in future observations and track how such information propagates to other unobserved nodes using red arrows. We have also expanded the caption for Figure 1 to better explain the figure.

Why does marginal benefit diminish with observations? Some explanation could have helped.

Response: A possible reason for the diminishing marginal benefit may be that additional observations can contain overlapping information that already exists in current observations. For instance, observing a neighbor of an existing observation may not provide much additional information. We have added an explanation in lines 291-295 in the revised manuscript.

**References**

1.	Arulampalam MS, Maskell S, Gordon N, Clapp T. A tutorial on particle filters for online nonlinear/non-Gaussian Bayesian tracking. IEEE Trans Signal Process. 2002;50: 174–188. doi:10.1109/78.978374

2.	Anderson JL. An Ensemble Adjustment Kalman Filter for Data Assimilation. Mon Weather Rev. 2001;129: 2884–2903. doi:10.1175/1520-0493(2001)129<2884:AEAKFF>2.0.CO;2

3.	Evensen G. Data assimilation: the ensemble Kalman filter. Springer Science & Business Media; 2009.

4.	Snyder C, Bengtsson T, Bickel P, Anderson J. Obstacles to High-Dimensional Particle Filtering. Mon Weather Rev. 2008;136: 4629–4640. doi:10.1175/2008MWR2529.1

5.	Pei S, Kandula S, Yang W, Shaman J. Forecasting the spatial transmission of influenza in the United States. Proc Natl Acad Sci. 2018;115: 2752–2757. doi:10.1073/pnas.1708856115

6.	Mandel J, Cobb L, Beezley JD. On the convergence of the ensemble Kalman filter. Appl Math. 2011;56: 533–541. doi:10.1007/s10492-011-0031-2

7.	Schillings C, Stuart AM. Convergence analysis of ensemble Kalman inversion: the linear, noisy case. Appl Anal. 2018;97: 107–123. doi:10.1080/00036811.2017.1386784

8.	Calvello E, Reich S, Stuart AM. Ensemble Kalman Methods: A Mean Field Perspective. arXiv; 2022. Available: http://arxiv.org/abs/2209.11371

9.	Lokhov AY, Mézard M, Ohta H, Zdeborová L. Inferring the origin of an epidemic with a dynamic message-passing algorithm. Phys Rev E. 2014;90: 012801. doi:10.1103/PhysRevE.90.012801
